# Medically Unexplained Oropharyngeal Dysphagia at the University Hospital ENT Outpatient Clinic for Dysphagia: A Cross-Sectional Cohort Study

**DOI:** 10.1007/s00455-018-9912-9

**Published:** 2018-06-05

**Authors:** Rob J.C.G. Verdonschot, Laura W.J. Baijens, Sophie Vanbelle, Michelle Florie, Remco Dijkman, Irene P.M. Leeters, Bernd Kremer, Carsten Leue

**Affiliations:** 1 Department of Otorhinolaryngology, Head and Neck Surgery, Maastricht University Medical Center, PO Box 5800, 6202 AZ Maastricht, The Netherlands; 2 Emergency Department, Erasmus Medical Center, Rotterdam, The Netherlands; 3 School of Mental Health and Neurosciences (MHeNS), Maastricht University, Maastricht, The Netherlands; 4 GROW-School for Oncology and Developmental Biology, Maastricht University Medical Center, Maastricht, The Netherlands; 5 Department of Methodology and Statistics, CAPHRI, Maastricht University, Maastricht, The Netherlands; 6 Department of Psychiatry and Psychology, Maastricht University Medical Center, Maastricht, The Netherlands

**Keywords:** Dysphagia, Affective symptoms, Anxiety, Depression

## Abstract

Medically unexplained oropharyngeal dysphagia (MUNOD) is a rare condition. It presents without demonstrable abnormalities in the anatomy of the upper aero-digestive tract and/or swallowing physiology. This study investigates whether MUNOD is related to affective or other psychiatric conditions. The study included patients with dysphagic complaints who had no detectible structural or physiological abnormalities upon swallowing examination. Patients with any underlying disease or disorder that could explain the oropharyngeal dysphagia were excluded. All patients underwent a standardized examination protocol, with FEES examination, the Hospital Anxiety and Depression Scale (HADS), and the Dysphagia Severity Scale (DSS). Two blinded judges scored five different FEES variables. None of the 14 patients included in this study showed any structural or physiological abnormalities during FEES examination. However, the majority did show abnormal piecemeal deglutition, which could be a symptom of MUNOD. Six patients (42.8%) had clinically relevant symptoms of anxiety and/or depression. The DSS scores did not differ significantly between patients with and without affective symptoms. Affective symptoms are common in patients with MUNOD, and their psychiatric conditions could possibly be related to their swallowing problems.

## Introduction

Patients with swallowing problems are commonly seen at the otorhinolaryngology outpatient clinic. Their oropharyngeal dysphagia (OD) may be attributed to somatic etiologies such as head and neck cancer, progressive neurological disorders, or stroke [1–3]. These disorders may change the normal anatomy and/or disturb normal function of the upper aero-digestive tract and thereby hamper normal swallowing. Rarely, OD occurs without demonstrable abnormalities in the anatomy of the upper aero-digestive tract and/or swallowing physiology, prompting a diagnosis of medically unexplained oropharyngeal dysphagia (MUNOD) [4]. In the literature, this condition is known by various names: functional dysphagia, swallowing phobia, psychogenic dysphagia, or phagophobia [4]. A functional somatic disorder is defined as physical complaints or symptoms impairing normal function of the bodily process that are not attributable to an underlying structural disease [5]. Functional somatic disorders and comorbid anxiety and depression are both associated with increased severity of symptoms and greater illness burden [6]. Medical specialties tend to apply their own diagnostic labels to functional somatic disorders. Psychiatry uses the term somatic symptom disorder, while other specialties make their own specific diagnosis (e.g., irritable bowel syndrome (IBS), fibromyalgia (FM), functional dyspepsia (FD)) [5, 7]. In the field of mental health, patients with MUNOD are frequently diagnosed with a functional somatic disorder or rarely with phagophobia (fear of swallowing). According to the DSM-V classification, phagophobia belongs to the category of ‘specific phobias’ [7], whereby exposure to the phobic stimulus provokes an immediate anxiety response. The phobic situation is avoided or endured with intense distress. Also, the specific phobia interferes with a patient’s normal routine, functioning, or social activities. Phagophobia can only be diagnosed if other psychiatric or somatic conditions are excluded as a possible cause for the dysphagia and accompanying emotional and bodily distress [7]. Patients with phagophobia experience an abnormal sensation during swallowing, sometimes accompanied by behavioral abnormalities during swallowing examination [7]. In the literature, phagophobia is often described in children [8, 9], but little is known about this condition in adults. Given the strong association of medically unexplained symptoms with affective conditions, it is advisable to use the broader term ‘MUNOD’ (instead of ‘phagophobia’). It may be a symptom within other psychiatric conditions like obsessive-compulsive disorder, panic disorder, post-traumatic stress disorder (PTSD), social phobia, or depression [10]. In patients with persistent complaints of MUNOD who do not show detectible abnormalities upon swallowing examination performed with fiberoptic endoscopic evaluation of swallowing (FEES) or videofluoroscopic swallowing study (VFSS), and who do not present with an underlying somatic disease, a possible cause of the complaints should be sought in a psychiatric condition (e.g., somatic symptom disorder, phagophobia, affective disorder, PTSD) [3, 4, 11, 12]. In most complex and high-utilizing patients with OD, affective or somatoform comorbidity should therefore be considered [13, 14].

### Aim

So far, no other studies have investigated whether patients with MUNOD have clinically relevant symptoms of anxiety and depression. This study is the first to inquire whether MUNOD is related to an affective condition or presents as a symptom within another psychiatric condition. The aim of this study is to better understand the psychiatric symptoms in patients with MUNOD and to provide guidance for integrated (otorhinolaryngological and psychiatric) management strategies in the context of best clinical practice.

## Materials and Methods

### Patients

Patients with OD complaints (usually choking) who were referred to the outpatient clinic for dysphagia of the Maastricht University Medical Center (MUMC+) between July 2011 and April 2016, without detectible abnormalities in swallowing examination, were included in the study. The following exclusion criteria were applied: age younger than 18, age older than 85 (presbyphagia), complaints of esophageal dysphagia (e.g., swallowing-related chest pain, esophageal regurgitation, history of esophageal dysphagia), history of head and neck cancer, evidence or suspicion of neurodegenerative disease (e.g., Myasthenia Gravis, multiple sclerosis, Parkinson’s disease), stroke patients, patients with a Zenker’s diverticulum or cervical spine abnormalities, patients with any other somatic disease or disorder that could explain the OD complaints, a score below 23 on the Mini Mental State Examination (MMSE) [15], or not knowing the Dutch language. Informed consent was obtained from all patients.

### Examination Protocol

All patients underwent a standardized examination protocol (prospectively collected data) used in daily clinical practice at the outpatient clinic for dysphagia. This protocol comprises a structured interview, standardized otorhinolaryngology examination, a standardized FEES examination [16], the Hospital Anxiety and Depression Scale (HADS) [17], a dysphagia severity scale (DSS) [14, 18], Body Mass Index (BMI) measurement, and the MMSE [15]. The FEES-examinations were carried out by an experienced laryngologist together with the speech therapist. First, patients had to perform three swallows of 10 cc thin liquid (water), then three swallows of 10 cc standardized applesauce (One 2 fruit^®^) (hereafter ‘thick liquid’), and then one bite-sized cracker (80 gr Delhaize Mini Toast^®^). All liquids were dyed with 5% methylene blue (10 mg/ml). A flexible fiberoptic endoscope, Pentax FNL-10RP3 (Pentax Canada Inc., Mississauga, Ontario, Canada), was used during the FEES examination. The tip of the endoscope was in ‘high position,’ just above the epiglottis, so the scope could not interfere with closure of the laryngeal vestibule [16]. The FEES videos were obtained with the Xion SD camera, Xion EndoSTROBE camera control unit (PAL 25 fps), and Matrix DS data station with DIVAS software (Xion Medical, Berlin, Germany) and recorded on a DVD. Second, the investigators administered the HADS, a validated tool to assess clinically relevant symptoms of anxiety and/or depression. It consists of 14 items: seven on the anxiety subscale and seven on the depression subscale. Each single item is scored from 0 to 3, resulting in a minimum of 0 and a maximum of 21 points on each subscale. A higher score indicates more anxiety or depression symptoms. A score of ≥ 8 on a subscale implies the presence of clinically relevant anxiety or depression symptoms, which is an indicator of an anxiety disorder or depression [17, 19, 20]. Third, a patient’s subjective swallowing assessment was measured with the DSS, a visual analog scale (VAS); this instrument is a psychometric response scale for measuring subjective characteristics or attitudes [14, 18]. Dysphagic patients specify their level of agreement with a statement or question by indicating a position along a continuous line between two end-points for the DSS. The single question was, “How do you rate your swallowing today?” A score of 100 (maximum) indicates normal swallowing. The MMSE is a tool to screen patient’s cognitive status. A score below 23 is interpreted as mild cognitive impairment for which a formal cognitive assessment to determine the pattern and extent of deficits is recommended. Therefore, to reduce possible bias in the HADS and DSS outcomes due to cognitive dysfunctions in the present study, patients with an MMSE below 23 were excluded.

### FEES Variables

To be sure that none of the selected patients had severe abnormalities during FEES examination (e.g., severe pooling, deep penetration, aspiration), suggesting a possible underlying somatic cause, five visuoperceptual ordinal variables (piecemeal deglutition, postswallow vallecular pooling, postswallow pyriform sinus pooling, laryngeal penetration, and aspiration) were scored by two independent judges [13, 21–26]. All of these variables were scored for every FEES swallow at varying speed. The judges underwent consensus training for these measurements, as described previously [13, 21–26]. Both judges were blinded to the patients’ identity and medical history. The judges were also blinded to each other’s scores. To determine intraobserver agreement, 30 (29%) of the FEES swallows were rated twice (repeated measurements). These FEES swallows were randomly selected and again blinded for both judges. Fatigue-related observer bias was avoided by limiting the judge’s rating task to two hours per session.

### Statistical Analysis

Levels of interobserver and intraobserver agreement were measured for each variable by the linear weighted kappa coefficient. Results were expressed as the median (range) for continuous variables, while frequencies and proportions (%) were used for ordinal FEES variables. The Mann–Whitney *U* test and the Chi-squared test were used for group comparisons. Spearman’s rho was used for correlations between continuous variables. All statistical analyses were performed with IBM SPSS Statistics for Mac, version 22.0 (Armonk, NY: IBM Corp.).

## Results

### Participants

Approximately 120 patients per year visited the outpatient clinic for dysphagia. Patients were referred by general practitioners, otorhinolaryngologists, or other specialists such as a neurologist or pulmonologist. The main reason for referral was to exclude pathology of the upper aero-digestive tract as a cause for OD. Fourteen patients met the criteria for MUNOD and were included in the study. The median age was 52 (19–68). In total seven of the participants (50%) were female. See Table 1 for general patients’ characteristics.

**Table 1:** Patients’ characteristics

Subject	Age	Gender	BMI	MMSE-score	Psychiatric history	Psychiatric medication	Referred by	No. of visits otorhinolaryngology outpatient clinic MUMC+
1	56	Female	29	30	–	–	GP	6
2	27	Male	17	30	Pervasive developmental disorder—not otherwise specified	–	Internist	1
3	43	Male	17	25	Cluster B personality disorder	Temazepam, Oxazepam	GP	2
4	41	Female	21	23	Panic disorder	Citalopram	Otorhinolaryngologist	3
5	51	Male	23	30	–	–	MV	1
6	68	Male	25	29	–	–	GP	1
7	26	Male	MV	23	–	–	GP	9
8	53	Male	MV	23	–	–	Otorhinolaryngologist	3
9	63	Female	MV	26	–	–	GP	1
10	19	Female	16	23	–	–	GP	1
11	60	Female	37	29	Psychotic depression	Quetiapine	Neurologist	1
12	61	Female	34	29	–	–	Internist	1
13	34	Female	20	30	–	–	GP	2
14	66	Male	25	30	–	–	Pulmonologist	2

*BMI* Body Mass Index, *MMSE* mini mental state examination, *GP* general practitioner, *MV* missing value, *MUMC+* Maastricht University Medical Center

### Observer Agreement

Table 2 shows levels of inter- and intraobserver agreement for all FEES variables with 95% confidence interval. Intraobserver agreement levels are shown for both raters separately. All levels of agreement were almost perfect (Kappa > 0.9). The lowest level of interobserver agreement was 0.95 (95% CI 0.89–1.00) for postswallow pyriform sinus pooling. The lowest level of intraobserver agreement was 0.90 (95% CI 0.80–1.00) for postswallow vallecular pooling. The prevalence of impairment was very low for all variables.

**Table 2:** Interobserver and intraobserver agreement levels per FEES variable assessed with linear weighted Kappa and 95% confidence interval

FEES outcome variable	Definition	Ordinal scale^a^	Interobserver agreement (95% CI)	Intraobserver agreement (95% CI)	
				Observer 1	Observer 2
Piecemeal deglutition	Sequential swallowing on the same bolus	Five-point scale (0–4) 0 = no additional swallows 1 = one additional swallow 2 = two additional swallows 3 = three additional swallows 4 = four additional swallows	0.99^b^ (0.97–1.00)	0.93 (0.84–1.00)	0.93 (0.84–1.00)
Postswallow vallecular pooling	Pooling in valleculae after the swallow	Three-point scale (0–2) 0 = no pooling 1 = filling of less than 50% of the valleculae 2 = filling of more than 50% of the valleculae	0.95 (0.91–1.00)	0.96 (0.89–1.00)	0.90 (0.80–1.00)
Postswallow pyriform sinus pooling	Pooling in pyriform sinuses after the swallow	Three-point scale (0–2) 0 = no pooling 1 = trace to moderate pooling 2 = severe pooling up to complete filling of the sinuses	0.95 (0.89–1.00)	1.00	1.00
Penetration and aspiration	Penetration of bolus in the laryngeal vestibule, above the vocal folds Aspiration of bolus below the vocal folds	Three-point scale (0–2) 0 = no penetration 1 = penetration 2= aspiration	0.98 (0.96–1.00)	0.97 (0.90–1.00)	0.97 (0.90–1.00)

Results of intraobserver agreement are given for both observers

^a^Lower scores refer to normal functioning, whereas higher scores refer to more severe disability

^b^Kappa values: < 0 = less than chance agreement; 1 = perfect agreement

### FEES Variables

Descriptive data of the FEES variables are displayed in Table 3. Piecemeal deglutition was rated as normal (category 0) in 31.0% (*N *= 13), 16.7% (*N *= 7), and 7.1% (*N* = 1) of the swallows for thin liquid consistency, thick liquid consistency, and bite-sized cracker, respectively. In five patients, postswallow vallecular pooling was rated as mild (14.3 and 7.1% of the swallows for thin liquid and thick liquid consistency, respectively), but in none of these patients was pooling observed in all seven recorded swallows. All five patients showed at least one normal swallow without vallecular pooling. None of the swallows was rated as severe vallecular pooling (category 2). Penetration was observed in two patients. The first patient showed a trace of methylene blue on the laryngeal side of the epiglottis during the first thin liquid swallow. The second patient showed deeper penetration, near the vocal folds, in multiple swallows and was therefore excluded because an underlying somatic cause of OD could not be excluded. None of the patients showed aspiration or pyriform sinus pooling during the swallowing examination. The study population was too small to perform further statistical analyses.

**Table 3:** Frequency distribution of swallows per category of the different FEES variables, given as absolute numbers (*N*) and percentages (%)

FEES category frequencies			
	Thin liquid consistency *N* (%) *N *= 42	Thick liquid consistency *N* (%) *N *= 42	Bite-sized cracker *N* (%) *N *= 14
Piecemeal deglutition			
Category 0	13 (31.0)	7 (16.7)	1 (7.1)
Category 1	10 (23.8)	15 (35.7)	2 (14.3)
Category 2	13 (30.9)	10 (23.8)	2 (14.3)
Category 3	1 (2.4)	3 (7.1)	2 (14.3)
Category 4	5 (11.9)	6 (14.3)	6 (42.9)
MV^a^	0	1 (2.4)	1 (7.1)
Postswallow vallecular pooling			
Category 0	35 (83.3)	36 (85.7)	12 (85.7)
Category 1	6 (14.3)	3 (7.1)	0
Category 2	0	0	0
MV	1 (2.4)	3 (7.1)	2 (14.3)
Postswallow pyriform sinus pooling			
Category 0	41 (97.6)	40 (95.2)	12 (85.7)
Category 1	0	0	0
Category 2	0	0	0
MV	1(2.4)	2 (4.8)	2 (14.3)
Penetration/aspiration			
Category 0	40 (95.2)	41 (97.6)	12 (85.7)
Category 1	1 (2.4)	0	0
Category 2	0	0	0
MV	1 (2.4)	1 (2.4)	2 (14.3)

^a^Missing value; FEES variable could not be rated

### Hads

Six of the 14 participants (42.8%) showed clinically relevant symptoms of anxiety (score ≥ 8 on the anxiety subscale). Three of the 14 (21.4%) showed clinically relevant symptoms of depression (score ≥ 8 on the depression subscale). These three also had a score ≥ 8 on the anxiety subscale. Thus, 42.8% (*N *= 6) of the participants had clinically relevant symptoms of anxiety and/or depression. The Chi-squared test showed no gender differences between patients with and without clinically relevant symptoms of anxiety (*p *= 0.28) or depression (*p *= 0.51). The Mann–Whitney *U* test showed no age differences between patients with and without clinically relevant symptoms of anxiety (*p *= 1.00) or depression (*p *= 0.76).

### DSS

The median score for the DSS was 66.0 (18–100). Spearman’s rho revealed no significant correlation between age and DSS. The DSS was not significantly different for patients with clinically relevant symptoms of anxiety or depression compared to patients without symptoms of anxiety or depression. Males scored significant higher on the DSS compared to females. See Table 4 for the results of the Mann–Whitney *U* tests for group comparison.

**Table 4:** Comparison of DSS between patients with clinically relevant symptoms of anxiety or depression and patients without symptoms of anxiety or depression using Mann–Whitney *U* test and comparison of DSS between male and female patients using the Mann–Whitney *U* test

	*N*	DSS score Median (range)	Level of significance *p*-value
HADS-D ≥ 8	3	85.0 (18–100)	0.659
HADS-D < 8	11	57.0 (31–98)	
HADS-A ≥ 8	6	76.0 (18–100)	0.662
HADS-A < 8	8	55.5 (31–98)	
Male	7	85.0 (44–100)	**0.017** ^a^
Female	7	54.0 (18–77)	

*DSS* Dysphagia Severity Score, *HADS-D* Depression subscale of the Hospital Anxiety and Depression Scale, *HADS-A* Anxiety subscale of the Hospital Anxiety and Depression Scale

^a^Statistically significant

## Discussion

This is the first study that investigates swallowing function in relation to symptoms of anxiety and depression in patients with MUNOD. All 14 included patients presented with complaints of OD, and none showed structural abnormalities during FEES examination. However, the majority showed abnormal piecemeal deglutition, which could be an early symptom of an underlying somatic disorder impairing normal swallowing physiology. However, it is conceivable that abnormal piecemeal deglutition is a clinically relevant symptom of MUNOD. Since these patients are often anxious about swallowing, multiple swallows of smaller fragments of the same bolus may offer them a sense of safety or control. In these patients, piecemeal deglutition seems to be a habitual coping strategy rather than a subclinical neurogenic impaired swallowing pattern. Nevertheless, follow-up for a possible progressive neurologic disease is recommended. It is assumed that swallowing physiology in patients with MUNOD is normal. However, an interesting question is whether MUNOD could disturb normal swallowing physiology. Roland et al. evaluated the incidence of esophageal contractility disturbances in psychiatric patients [27]. Manometry showed a high percentage of functional motor impairment in patients with complaints of anxiety and/or depression, while endoscopy in these patients showed no structural abnormalities [27]. In a large prospective population-based study, Koloski et al. showed that anxiety is an independent predictor for new onset functional gastrointestinal disorders like irritable bowel syndrome, suggesting that affective disorders can underlie physical symptoms [28]. The bladder–gut–brain axis is an interesting framework. It suggests a bidirectional pathway between brain and body, assuming that both functional and affective disorders are stress related and that functional symptoms are a sensitized response to earlier threats. This sensitization might mediate false-alarm signals (alarm falsification as a defense system). That, in turn, could provoke emotional and physical distress, resulting in psychiatric conditions and functional disorders like MUNOD [6, 28]. A study by Dum et al. raised the possibility that motor areas of the cerebral cortex are important in the stress and depression connectome [29], and Grillon et al. suggested that anxiety increases motor response inhibition [30]. These studies indicate a relationship between affective function and motor function and thus strengthen the assumption that functional complaints might be part of a hypersensitivity or alarm-falsification disorder [6]. By implication, MUNOD and functional motor impairment may be interrelated too, causing disturbances of the normal swallowing physiology (such as increased piecemeal deglutition). So far, no studies have been published on this subject. However, the assumption that patients with MUNOD must have a normal swallowing function might be incorrect. Through this bidirectional pathway, a psychiatric problem can have sensorimotor effects on the swallowing function without there being any other cause of dysphagia, such as a chronic neurological disorder. Then, it would be plausible that OD can be caused by affective disorders or psychiatric conditions, even when the swallowing physiology is disturbed. In this study, none of the participants had symptoms indicating an underlying somatic disease, and none showed other abnormalities during structured interviews or general otorhinolaryngology examination (normal cranial nerve integrity, speech, etc.). Although a somatic cause of dysphagia might seem unlikely, MUNOD should always be a diagnosis of exclusion.

Previous research showed a high prevalence of clinically relevant affective symptoms in OD patients [13, 14, 31]. The present study underpins these data. It also shows a high prevalence (42.8%) of clinically relevant affective symptoms, which indicates that MUNOD seems to be related to affective conditions in more than 40% of the cases. Four of the participants (28.5%) had already been diagnosed with a psychiatric condition (psychotic depression, panic disorder, pervasive developmental disorder—not otherwise specified, cluster B personality disorder). The patient with cluster B personality disorder showed clinically relevant symptoms of anxiety and depression, and the patient with panic disorder exhibited clinically relevant symptoms of anxiety. In these patients, MUNOD and affective symptoms are likely to be part of their psychiatric disorder.

The DSS scores were not significantly different between patients with and without clinically relevant affective symptoms. Apparently, clinically relevant symptoms of anxiety and depression are not related to the severity of MUNOD symptoms. A psychological screening questionnaire, like the HADS, is a simple tool for the preliminary assessment of the affective state of a patient. However, the expertise of a psychiatrist is essential to a definitive diagnosis and treatment of any psychiatric condition, including phagophobia or other anxiety disorders, and depression. It might be helpful to draw upon the patient’s psychiatric history and to involve his or her own psychiatrist when preparing a multidisciplinary treatment strategy. Involvement of a psychiatrist would obviously be necessary. However, the patient must be willing to cooperate and accept that a psychiatric problem might be the cause of the swallowing problems. In this study, only four patients could be convinced to visit a psychiatrist after visiting the outpatient clinic for dysphagia. Following referral to the psychiatrist, one patient was diagnosed with an anxiety disorder and one patient was diagnosed with an identity disorder. Two of the referred patients were already known with a psychiatric disorder (panic disorder and psychotic depression), see Table 1. Early recognition of MUNOD and a motivational trajectory towards integrated care are necessary to develop effective treatment strategies, to reduce health care consumption and health care costs, to decrease the risk of iatrogenic damage arising from continuous diagnostic intervention, and to prevent frustration in the interaction between physician and patient [11]. Almost all of the participants had already consulted multiple specialists or had made recurrent visits to outpatient clinics all over the Netherlands. Consultation of a psychiatrist must be considered as an early option in the diagnostic strategy of MUNOD instead of the ‘last resort’ after unsuccessful treatment. Diagnosis and treatment of an underlying psychiatric disease may improve the swallowing problems. It is important to realize that affective symptoms are frequently present in patients with MUNOD. Assuming a bidirectional pathway between brain and body, MUNOD could be understood as a symptom of physical distress or part of an alarm falsification and defense reaction as seen in other functional syndromes. In patients with prolonged dysphagic complaints, with no indication of a somatic disease or abnormality, psychiatric conditions must be considered as a possible cause of OD. Validated psychological screening questionnaires could be helpful in the detection of affective conditions but also of other psychiatric conditions. Involvement of a psychiatrist and/or psychologist is recommended.

### Limitations of the Study

This investigation has some limitations. First, since MUNOD is a rare condition, the number of patients included in the study is small, so only a limited statistical analysis could be performed. Second, the HADS questionnaire was used for screening of anxiety and depression symptoms. Possibly, a different screening tool or multiple screening tools would have led to different results. Third, three of the participants were taking psychiatric medication (see Table 1), which could have a negative effect on swallowing [32, 33]. Furthermore, the use of psychiatric medication could have led to an underestimation of the HADS scores. Furthermore, this investigation used a cross-sectional study design and was not intended as a therapy-effect study; the effect of different treatment options could be examined in future research, which could also specify treatment strategies in patients with MUNOD and psychiatric comorbidity.

## Conclusion

MUNOD is a rare condition that is difficult to diagnose. We hope to help dysphagia caregivers by sharing our results and experiences. Patients deserve a professional approach, particularly because their diagnostic trajectory has often been long and inconclusive. Affective symptoms are common in these patients. MUNOD could be a symptom of a psychiatric condition or part of the alarm falsification defense system, suggesting that physical symptoms and affective disorders are stress-related and a response to earlier threats. Consultation of a psychiatrist for patients with MUNOD is recommended as part of a pathway toward multidisciplinary integrated care.
